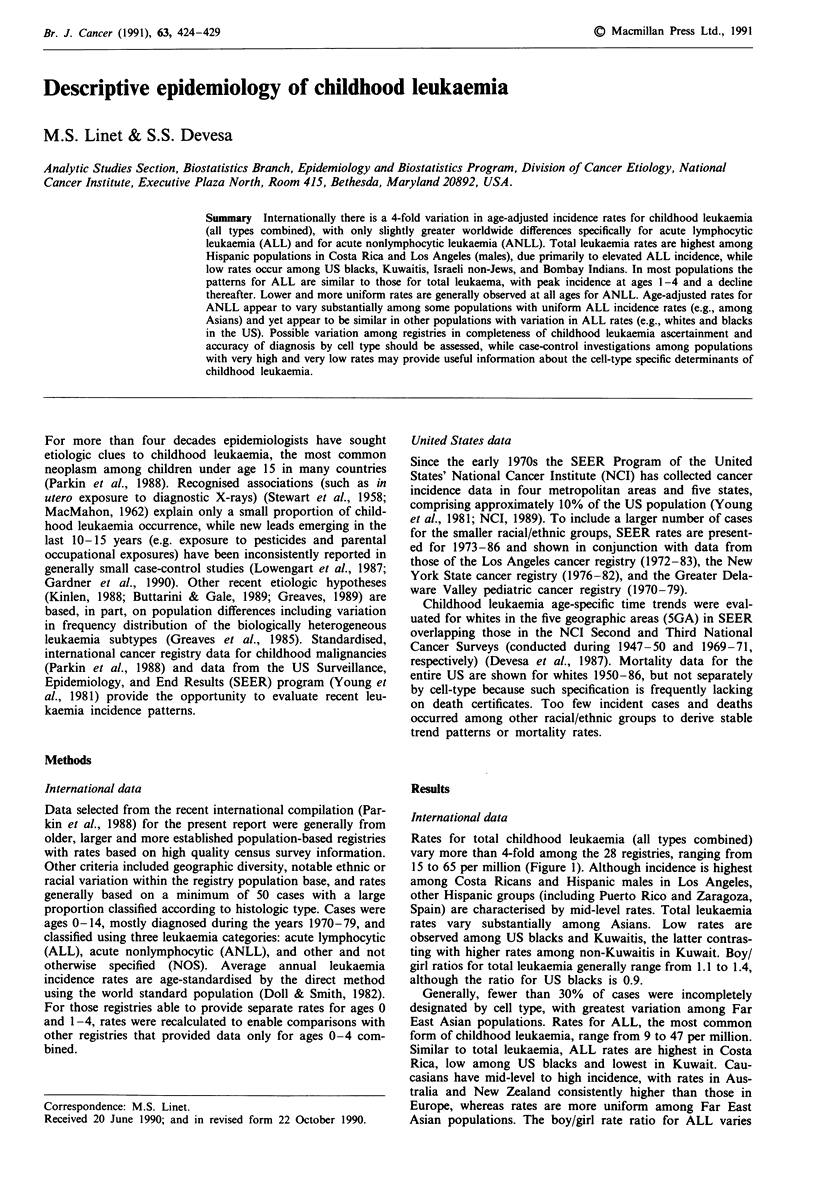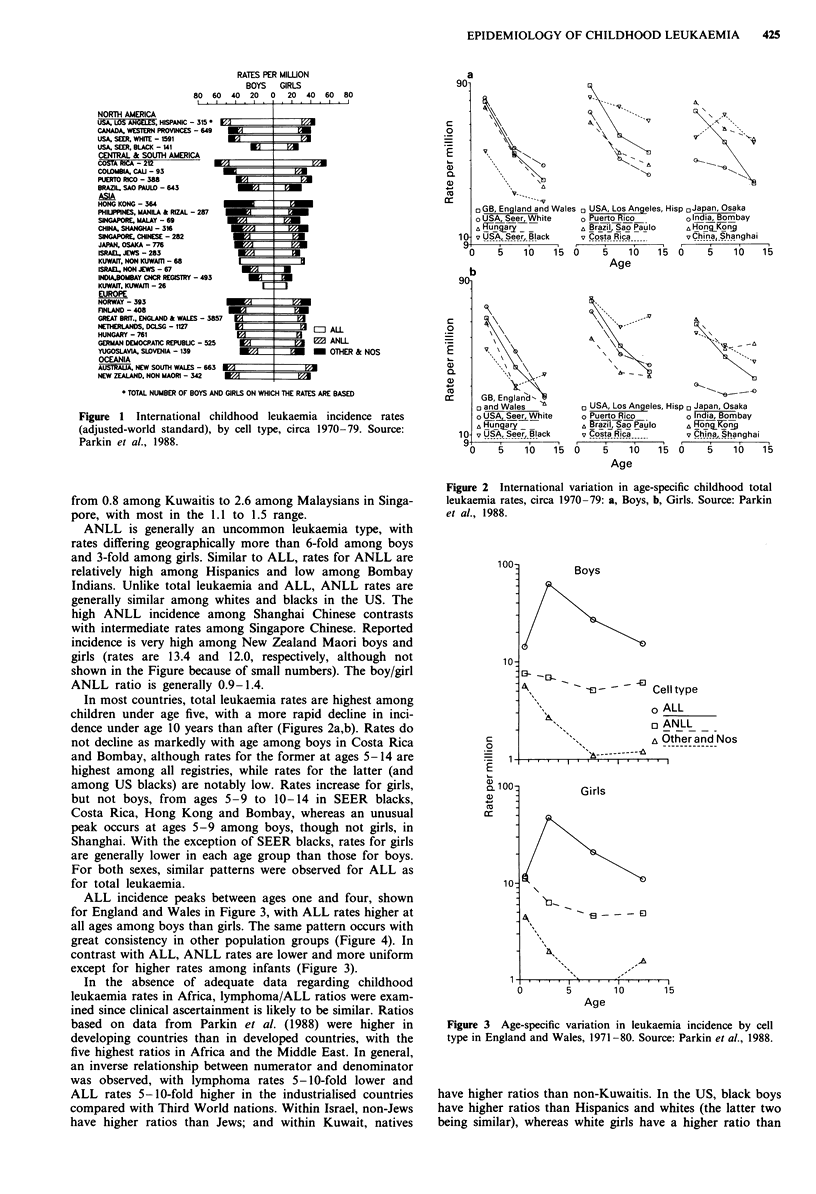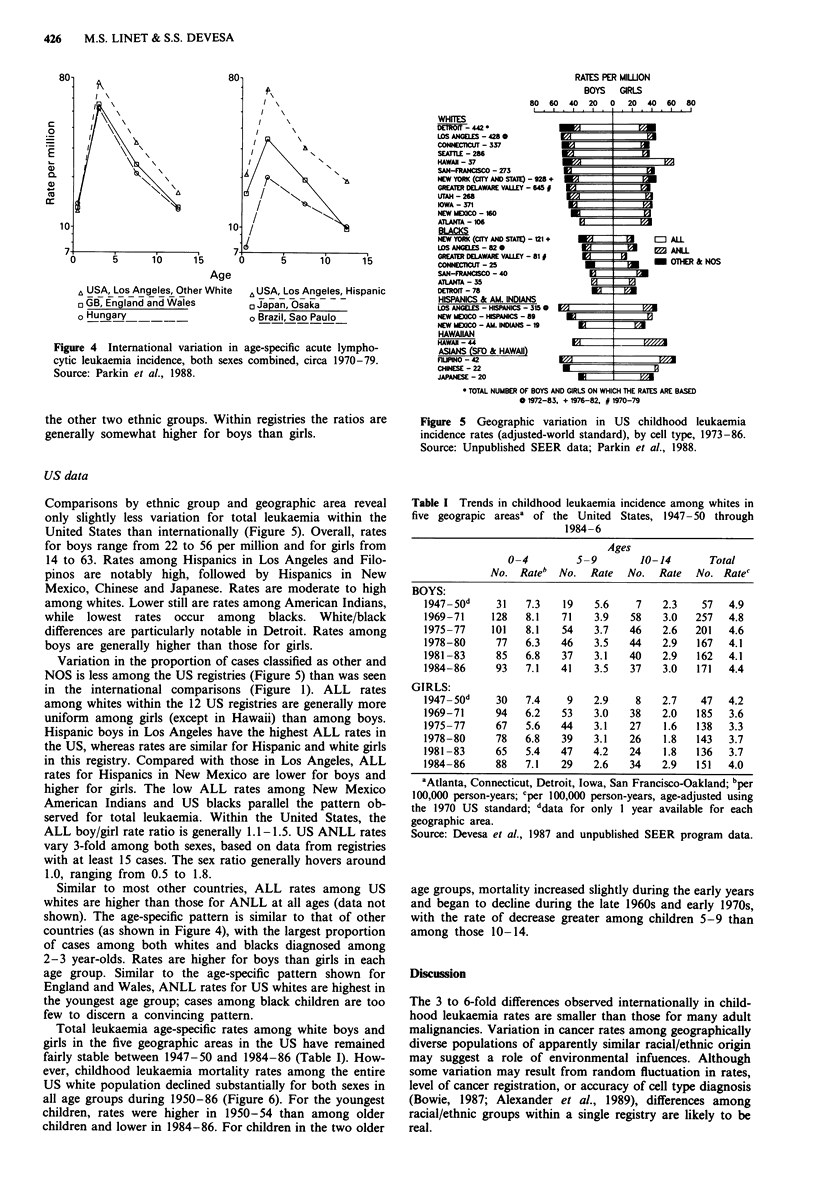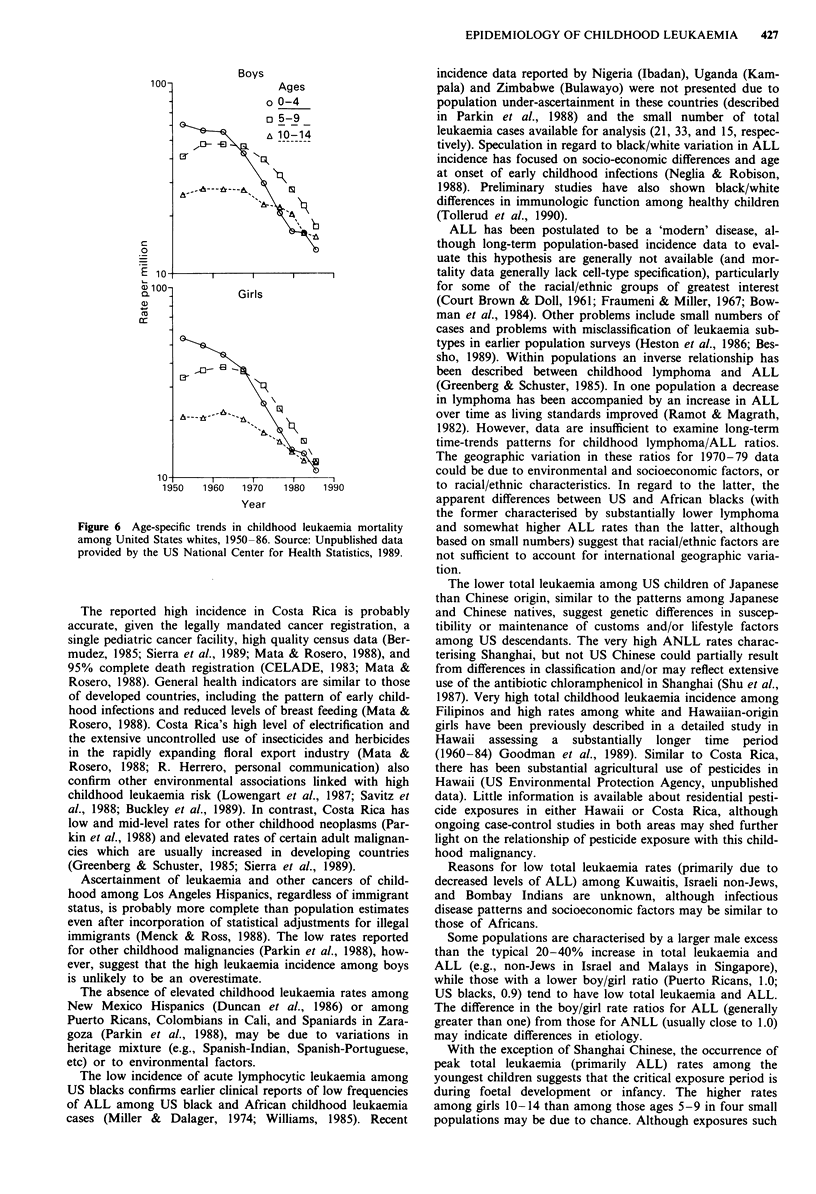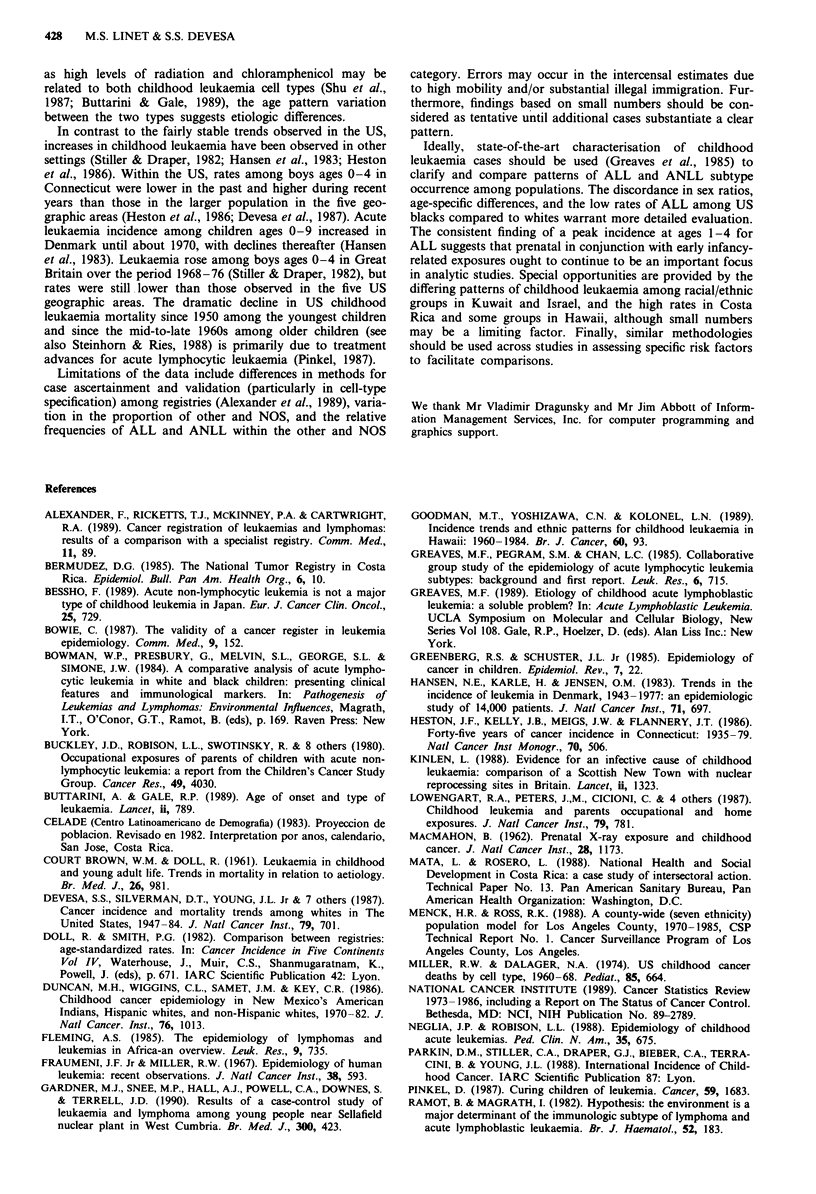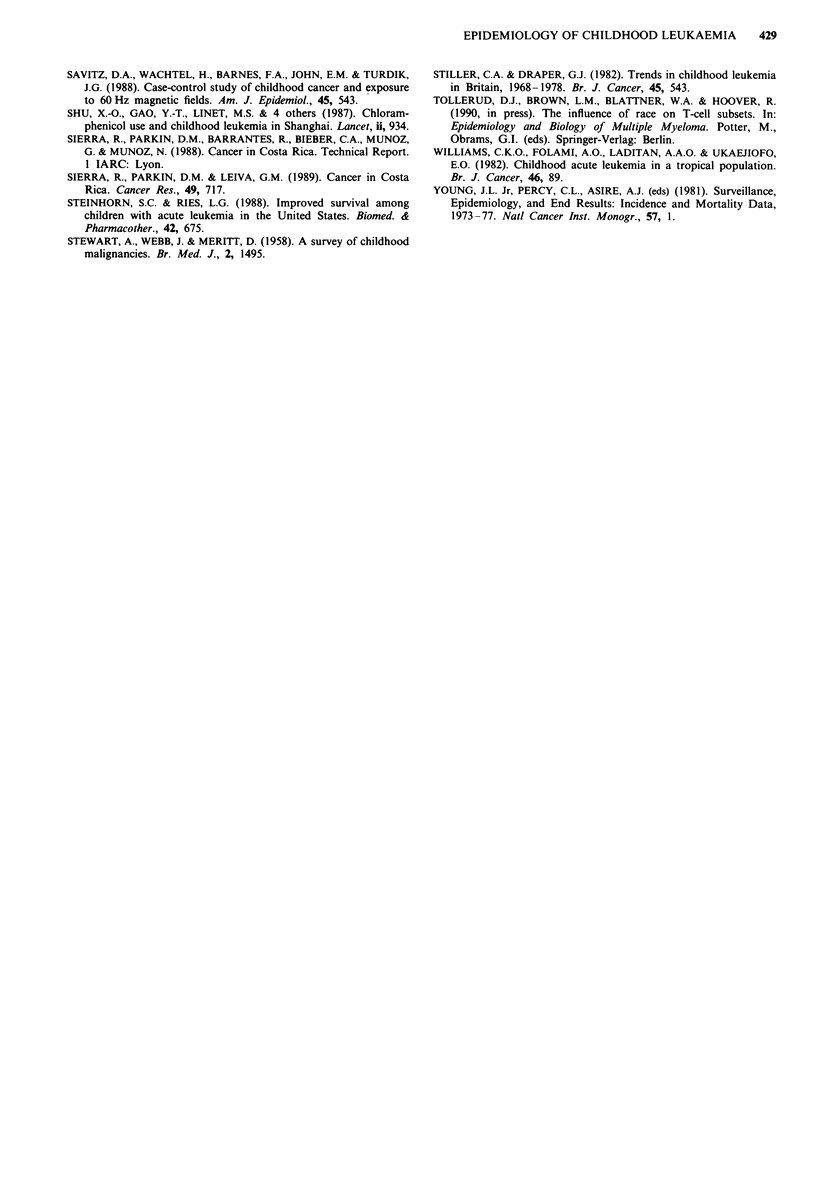# Descriptive epidemiology of childhood leukaemia.

**DOI:** 10.1038/bjc.1991.98

**Published:** 1991-03

**Authors:** M. S. Linet, S. S. Devesa

**Affiliations:** Analytic Studies Section, National Cancer Institute, Bethesda, Maryland 20892.

## Abstract

Internationally there is a 4-fold variation in age-adjusted incidence rates for childhood leukaemia (all types combined), with only slightly greater worldwide differences specifically for acute lymphocytic leukaemia (ALL) and for acute nonlymphocytic leukaemia (ANLL). Total leukaemia rates are highest among Hispanic populations in Costa Rica and Los Angeles (males), due primarily to elevated ALL incidence, while low rates occur among US blacks, Kuwaitis, Israeli non-Jews, and Bombay Indians. In most populations the patterns for ALL are similar to those for total leukaema, with peak incidence at ages 1-4 and a decline thereafter. Lower and more uniform rates are generally observed at all ages for ANLL. Age-adjusted rates for ANLL appear to vary substantially among some populations with uniform ALL incidence rates (e.g., among Asians) and yet appear to be similar in other populations with variation in ALL rates (e.g., whites and blacks in the US). Possible variation among registries in completeness of childhood leukaemia ascertainment and accuracy of diagnosis by cell type should be assessed, while case-control investigations among populations with very high and very low rates may provide useful information about the cell-type specific determinants of childhood leukaemia.


Br. J. Cancer (1991), 63, 424-429 © Macmillan Press Ltd., 1991

### Descriptive epidemiology of childhood leukaemia

M.S. Linet & S.S. Devesa

*Analytic Studies Section, Biostatistics Branch, Epidemiology and Biostatistics Program, Division of Cancer Etiology, National Cancer Institute, Executive Plaza North, Room 415, Bethesda, Maryland 20892, USA.*

**Summary** Internationally there is a 4-fold variation in age-adjusted incidence rates for childhood leukaemia (all types combined), with only slightly greater worldwide differences specifically for acute lymphocytic leukaemia (ALL) and for acute nonlymphocytic leukaemia (ANLL). Total leukaemia rates are highest among Hispanic populations in Costa Rica and Los Angeles (males), due primarily to elevated ALL incidence, while low rates occur among US blacks, Kuwaitis, Israeli non-Jews, and Bombay Indians. In most populations the patterns for ALL are similar to those for total leukaema, with peak incidence at ages 1-4 and a decline thereafter. Lower and more uniform rates are generally observed at all ages for ANLL. Age-adjusted rates for ANLL appear to vary substantially among some populations with uniform ALL incidence rates (e.g., among Asians) and yet appear to be similar in other populations with variation in ALL rates (e.g., whites and blacks in the US). Possible variation among registries in completeness of childhood leukaemia ascertainment and accuracy of diagnosis by cell type should be assessed, while case-control investigations among populations with very high and very low rates may provide useful information about the cell-type specific determinants of childhood leukaemia.

For more than four decades epidemiologists have sought etiologic clues to childhood leukaemia, the most common neoplasm among children under age 15 in many countries (Parkin *et al.*, 1988). Recognised associations (such as *in utero* exposure to diagnostic X-rays) (Stewart *et al.*, 1958; MacMahon, 1962) explain only a small proportion of childhood leukaemia occurrence, while new leads emerging in the last 10-15 years (e.g. exposure to pesticides and parental occupational exposures) have been inconsistently reported in generally small case-control studies (Lowengart *et al.*, 1987; Gardner *et al.*, 1990). Other recent etiologic hypotheses (Kinlen, 1988; Buttarini & Gale, 1989; Greaves, 1989) are based, in part, on population differences including variation in frequency distribution of the biologically heterogeneous leukaemia subtypes (Greaves *et al.*, 1985). Standardised, international cancer registry data for childhood malignancies (Parkin *et al.*, 1988) and data from the US Surveillance, Epidemiology, and End Results (SEER) program (Young *et al.*, 1981) provide the opportunity to evaluate recent leukaemia incidence patterns.

### Methods

### *International data*

Data selected from the recent international compilation (Parkin *et al.*, 1988) for the present report were generally from older, larger and more established population-based registries with rates based on high quality census survey information. Other criteria included geographic diversity, notable ethnic or racial variation within the registry population base, and rates generally based on a minimum of 50 cases with a large proportion classified according to histologic type. Cases were ages 0-14, mostly diagnosed during the years 1970-79, and classified using three leukaemia categories: acute lymphocytic (ALL), acute nonlymphocytic (ANLL), and other and not otherwise specified (NOS). Average annual leukaemia incidence rates are age-standardised by the direct method using the world standard population (Doll & Smith, 1982). For those registries able to provide separate rates for ages 0 and 1-4, rates were recalculated to enable comparisons with other registries that provided data only for ages 0-4 combined.

### *United States data*

Since the early 1970s the SEER Program of the United States' National Cancer Institute (NCI) has collected cancer incidence data in four metropolitan areas and five states, comprising approximately 10% of the US population (Young *et al.*, 1981; NCI, 1989). To include a larger number of cases for the smaller racial/ethnic groups, SEER rates are presented for 1973-86 and shown in conjunction with data from those of the Los Angeles cancer registry (1972-83), the New York State cancer registry (1976-82), and the Greater Delaware Valley pediatric cancer registry (1970-79).

Childhood leukaemia age-specific time trends were evaluated for whites in the five geographic areas (5GA) in SEER overlapping those in the NCI Second and Third National Cancer Surveys (conducted during 1947-50 and 1969-71, respectively) (Devesa *et al.*, 1987). Mortality data for the entire US are shown for whites 1950-86, but not separately by cell-type because such specification is frequently lacking on death certificates. Too few incident cases and deaths occurred among other racial/ethnic groups to derive stable trend patterns or mortality rates.

### Results

### *International data*

Rates for total childhood leukaemia (all types combined) vary more than 4-fold among the 28 registries, ranging from 15 to 65 per million (Figure 1). Although incidence is highest among Costa Ricans and Hispanic males in Los Angeles, other Hispanic groups (including Puerto Rico and Zaragoza, Spain) are characterised by mid-level rates. Total leukaemia rates vary substantially among Asians. Low rates are observed among US blacks and Kuwaitis, the latter contrasting with higher rates among non-Kuwaitis in Kuwait. Boy/girl ratios for total leukaemia generally range from 1.1 to 1.4, although the ratio for US blacks is 0.9.

Generally, fewer than 30% of cases were incompletely designated by cell type, with greatest variation among Far East Asian populations. Rates for ALL, the most common form of childhood leukaemia, range from 9 to 47 per million. Similar to total leukaemia, ALL rates are highest in Costa Rica, low among US blacks and lowest in Kuwait. Caucasians have mid-level to high incidence, with rates in Australia and New Zealand consistently higher than those in Europe, whereas rates are more uniform among Far East Asian populations. The boy/girl rate ratio for ALL varies

Correspondence: M.S. Linet.
Received 20 June 1990; and in revised form 22 October 1990.

**Figure 1** International childhood leukaemia incidence rates (adjusted-world standard), by cell type, circa 1970–79. Source: Parkin *et al.*, 1988.

**Figure 2** International variation in age-specific childhood total leukaemia rates, circa 1970–79: **a**, Boys, **b**, Girls. Source: Parkin *et al.*, 1988.

from 0.8 among Kuwaitis to 2.6 among Malaysians in Singapore, with most in the 1.1 to 1.5 range.

ANLL is generally an uncommon leukaemia type, with rates differing geographically more than 6-fold among boys and 3-fold among girls. Similar to ALL, rates for ANLL are relatively high among Hispanics and low among Bombay Indians. Unlike total leukaemia and ALL, ANLL rates are generally similar among whites and blacks in the US. The high ANLL incidence among Shanghai Chinese contrasts with intermediate rates among Singapore Chinese. Reported incidence is very high among New Zealand Maori boys and girls (rates are 13.4 and 12.0, respectively, although not shown in the Figure because of small numbers). The boy/girl ANLL ratio is generally 0.9–1.4.

In most countries, total leukaemia rates are highest among children under age five, with a more rapid decline in incidence under age 10 years than after (Figures 2a,b). Rates do not decline as markedly with age among boys in Costa Rica and Bombay, although rates for the former at ages 5–14 are highest among all registries, while rates for the latter (and among US blacks) are notably low. Rates increase for girls, but not boys, from ages 5–9 to 10–14 in SEER blacks, Costa Rica, Hong Kong and Bombay, whereas an unusual peak occurs at ages 5–9 among boys, though not girls, in Shanghai. With the exception of SEER blacks, rates for girls are generally lower in each age group than those for boys. For both sexes, similar patterns were observed for ALL as for total leukaemia.

ALL incidence peaks between ages one and four, shown for England and Wales in Figure 3, with ALL rates higher at all ages among boys than girls. The same pattern occurs with great consistency in other population groups (Figure 4). In contrast with ALL, ANLL rates are lower and more uniform except for higher rates among infants (Figure 3).

In the absence of adequate data regarding childhood leukaemia rates in Africa, lymphoma/ALL ratios were examined since clinical ascertainment is likely to be similar. Ratios based on data from Parkin *et al.* (1988) were higher in developing countries than in developed countries, with the five highest ratios in Africa and the Middle East. In general, an inverse relationship between numerator and denominator was observed, with lymphoma rates 5–10-fold lower and ALL rates 5–10-fold higher in the industrialised countries compared with Third World nations. Within Israel, non-Jews have higher ratios than Jews; and within Kuwait, natives

**Figure 3** Age-specific variation in leukaemia incidence by cell type in England and Wales, 1971–80. Source: Parkin *et al.*, 1988.

have higher ratios than non-Kuwaitis. In the US, black boys have higher ratios than Hispanics and whites (the latter two being similar), whereas white girls have a higher ratio than

**Figure 4** International variation in age-specific acute lymphocytic leukaemia incidence, both sexes combined, circa 1970–79. Source: Parkin *et al.*, 1988.

**Figure 5** Geographic variation in US childhood leukaemia incidence rates (adjusted-world standard), by cell type, 1973–86. Source: Unpublished SEER data; Parkin *et al.*, 1988.

the other two ethnic groups. Within registries the ratios are generally somewhat higher for boys than girls.

*US data*

Comparisons by ethnic group and geographic area reveal only slightly less variation for total leukaemia within the United States than internationally (Figure 5). Overall, rates for boys range from 22 to 56 per million and for girls from 14 to 63. Rates among Hispanics in Los Angeles and Filopinos are notably high, followed by Hispanics in New Mexico, Chinese and Japanese. Rates are moderate to high among whites. Lower still are rates among American Indians, while lowest rates occur among blacks. White/black differences are particularly notable in Detroit. Rates among boys are generally higher than those for girls.

Variation in the proportion of cases classified as other and NOS is less among the US registries (Figure 5) than was seen in the international comparisons (Figure 1). ALL rates among whites within the 12 US registries are generally more uniform among girls (except in Hawaii) than among boys. Hispanic boys in Los Angeles have the highest ALL rates in the US, whereas rates are similar for Hispanic and white girls in this registry. Compared with those in Los Angeles, ALL rates for Hispanics in New Mexico are lower for boys and higher for girls. The low ALL rates among New Mexico American Indians and US blacks parallel the pattern observed for total leukaemia. Within the United States, the ALL boy/girl rate ratio is generally 1.1–1.5. US ANLL rates vary 3-fold among both sexes, based on data from registries with at least 15 cases. The sex ratio generally hovers around 1.0, ranging from 0.5 to 1.8.

Similar to most other countries, ALL rates among US whites are higher than those for ANLL at all ages (data not shown). The age-specific pattern is similar to that of other countries (as shown in Figure 4), with the largest proportion of cases among both whites and blacks diagnosed among 2–3 year-olds. Rates are higher for boys than girls in each age group. Similar to the age-specific pattern shown for England and Wales, ANLL rates for US whites are highest in the youngest age group; cases among black children are too few to discern a convincing pattern.

Total leukaemia age-specific rates among white boys and girls in the five geographic areas in the US have remained fairly stable between 1947–50 and 1984–86 (Table I). However, childhood leukaemia mortality rates among the entire US white population declined substantially for both sexes in all age groups during 1950–86 (Figure 6). For the youngest children, rates were higher in 1950–54 than among older children and lower in 1984–86. For children in the two older age groups, mortality increased slightly during the early years and began to decline during the late 1960s and early 1970s, with the rate of decrease greater among children 5–9 than among those 10–14.

**Table I** Trends in childhood leukaemia incidence among whites in five geograpic areas[a] of the United States, 1947–50 through 1984–6

| | Ages | | | | | | | |
|---|---|---|---|---|---|---|---|---|
| | *0–4* | | *5–9* | | *10–14* | | *Total* | |
| | *No.* | *Rate*[b] | *No.* | *Rate* | *No.* | *Rate* | *No.* | *Rate*[c] |
| BOYS: | | | | | | | | |
| 1947–50[d] | 31 | 7.3 | 19 | 5.6 | 7 | 2.3 | 57 | 4.9 |
| 1969–71 | 128 | 8.1 | 71 | 3.9 | 58 | 3.0 | 257 | 4.8 |
| 1975–77 | 101 | 8.1 | 54 | 3.7 | 46 | 2.6 | 201 | 4.6 |
| 1978–80 | 77 | 6.3 | 46 | 3.5 | 44 | 2.9 | 167 | 4.1 |
| 1981–83 | 85 | 6.8 | 37 | 3.1 | 40 | 2.9 | 162 | 4.1 |
| 1984–86 | 93 | 7.1 | 41 | 3.5 | 37 | 3.0 | 171 | 4.4 |
| GIRLS: | | | | | | | | |
| 1947–50[d] | 30 | 7.4 | 9 | 2.9 | 8 | 2.7 | 47 | 4.2 |
| 1969–71 | 94 | 6.2 | 53 | 3.0 | 38 | 2.0 | 185 | 3.6 |
| 1975–77 | 67 | 5.6 | 44 | 3.1 | 27 | 1.6 | 138 | 3.3 |
| 1978–80 | 78 | 6.8 | 39 | 3.1 | 26 | 1.8 | 143 | 3.7 |
| 1981–83 | 65 | 5.4 | 47 | 4.2 | 24 | 1.8 | 136 | 3.7 |
| 1984–86 | 88 | 7.1 | 29 | 2.6 | 34 | 2.9 | 151 | 4.0 |

[a]Atlanta, Connecticut, Detroit, Iowa, San Francisco-Oakland; [b]per 100,000 person-years; [c]per 100,000 person-years, age-adjusted using the 1970 US standard; [d]data for only 1 year available for each geographic area.
Source: Devesa *et al.*, 1987 and unpublished SEER program data.

### Discussion

The 3 to 6-fold differences observed internationally in childhood leukaemia rates are smaller than those for many adult malignancies. Variation in cancer rates among geographically diverse populations of apparently similar racial/ethnic origin may suggest a role of environmental infuences. Although some variation may result from random fluctuation in rates, level of cancer registration, or accuracy of cell type diagnosis (Bowie, 1987; Alexander *et al.*, 1989), differences among racial/ethnic groups within a single registry are likely to be real.

**Figure 6** Age-specific trends in childhood leukaemia mortality among United States whites, 1950–86. Source: Unpublished data provided by the US National Center for Health Statistics, 1989.

The reported high incidence in Costa Rica is probably accurate, given the legally mandated cancer registration, a single pediatric cancer facility, high quality census data (Bermudez, 1985; Sierra *et al.*, 1989; Mata & Rosero, 1988), and 95% complete death registration (CELADE, 1983; Mata & Rosero, 1988). General health indicators are similar to those of developed countries, including the pattern of early childhood infections and reduced levels of breast feeding (Mata & Rosero, 1988). Costa Rica's high level of electrification and the extensive uncontrolled use of insecticides and herbicides in the rapidly expanding floral export industry (Mata & Rosero, 1988; R. Herrero, personal communication) also confirm other environmental associations linked with high childhood leukaemia risk (Lowengart *et al.*, 1987; Savitz *et al.*, 1988; Buckley *et al.*, 1989). In contrast, Costa Rica has low and mid-level rates for other childhood neoplasms (Parkin *et al.*, 1988) and elevated rates of certain adult malignancies which are usually increased in developing countries (Greenberg & Schuster, 1985; Sierra *et al.*, 1989).

Ascertainment of leukaemia and other cancers of childhood among Los Angeles Hispanics, regardless of immigrant status, is probably more complete than population estimates even after incorporation of statistical adjustments for illegal immigrants (Menck & Ross, 1988). The low rates reported for other childhood malignancies (Parkin *et al.*, 1988), however, suggest that the high leukaemia incidence among boys is unlikely to be an overestimate.

The absence of elevated childhood leukaemia rates among New Mexico Hispanics (Duncan *et al.*, 1986) or among Puerto Ricans, Colombians in Cali, and Spaniards in Zaragoza (Parkin *et al.*, 1988), may be due to variations in heritage mixture (e.g., Spanish-Indian, Spanish-Portuguese, etc) or to environmental factors.

The low incidence of acute lymphocytic leukaemia among US blacks confirms earlier clinical reports of low frequencies of ALL among US black and African childhood leukaemia cases (Miller & Dalager, 1974; Williams, 1985). Recent incidence data reported by Nigeria (Ibadan), Uganda (Kampala) and Zimbabwe (Bulawayo) were not presented due to population under-ascertainment in these countries (described in Parkin *et al.*, 1988) and the small number of total leukaemia cases available for analysis (21, 33, and 15, respectively). Speculation in regard to black/white variation in ALL incidence has focused on socio-economic differences and age at onset of early childhood infections (Neglia & Robison, 1988). Preliminary studies have also shown black/white differences in immunologic function among healthy children (Tollerud *et al.*, 1990).

ALL has been postulated to be a 'modern' disease, although long-term population-based incidence data to evaluate this hypothesis are generally not available (and mortality data generally lack cell-type specification), particularly for some of the racial/ethnic groups of greatest interest (Court Brown & Doll, 1961; Fraumeni & Miller, 1967; Bowman *et al.*, 1984). Other problems include small numbers of cases and problems with misclassification of leukaemia subtypes in earlier population surveys (Heston *et al.*, 1986; Bessho, 1989). Within populations an inverse relationship has been described between childhood lymphoma and ALL (Greenberg & Schuster, 1985). In one population a decrease in lymphoma has been accompanied by an increase in ALL over time as living standards improved (Ramot & Magrath, 1982). However, data are insufficient to examine long-term time-trends patterns for childhood lymphoma/ALL ratios. The geographic variation in these ratios for 1970–79 data could be due to environmental and socioeconomic factors, or to racial/ethnic characteristics. In regard to the latter, the apparent differences between US and African blacks (with the former characterised by substantially lower lymphoma and somewhat higher ALL rates than the latter, although based on small numbers) suggest that racial/ethnic factors are not sufficient to account for international geographic variation.

The lower total leukaemia among US children of Japanese than Chinese origin, similar to the patterns among Japanese and Chinese natives, suggest genetic differences in susceptibility or maintenance of customs and/or lifestyle factors among US descendants. The very high ANLL rates characterising Shanghai, but not US Chinese could partially result from differences in classification and/or may reflect extensive use of the antibiotic chloramphenicol in Shanghai (Shu *et al.*, 1987). Very high total childhood leukaemia incidence among Filipinos and high rates among white and Hawaiian-origin girls have been previously described in a detailed study in Hawaii assessing a substantially longer time period (1960–84) Goodman *et al.*, 1989). Similar to Costa Rica, there has been substantial agricultural use of pesticides in Hawaii (US Environmental Protection Agency, unpublished data). Little information is available about residential pesticide exposures in either Hawaii or Costa Rica, although ongoing case-control studies in both areas may shed further light on the relationship of pesticide exposure with this childhood malignancy.

Reasons for low total leukaemia rates (primarily due to decreased levels of ALL) among Kuwaitis, Israeli non-Jews, and Bombay Indians are unknown, although infectious disease patterns and socioeconomic factors may be similar to those of Africans.

Some populations are characterised by a larger male excess than the typical 20–40% increase in total leukaemia and ALL (e.g., non-Jews in Israel and Malays in Singapore), while those with a lower boy/girl ratio (Puerto Ricans, 1.0; US blacks, 0.9) tend to have low total leukaemia and ALL. The difference in the boy/girl rate ratios for ALL (generally greater than one) from those for ANLL (usually close to 1.0) may indicate differences in etiology.

With the exception of Shanghai Chinese, the occurrence of peak total leukaemia (primarily ALL) rates among the youngest children suggests that the critical exposure period is during foetal development or infancy. The higher rates among girls 10–14 than among those ages 5–9 in four small populations may be due to chance. Although exposures such as high levels of radiation and chloramphenicol may be related to both childhood leukaemia cell types (Shu *et al.*, 1987; Buttarini & Gale, 1989), the age pattern variation between the two types suggests etiologic differences.

In contrast to the fairly stable trends observed in the US, increases in childhood leukaemia have been observed in other settings (Stiller & Draper, 1982; Hansen *et al.*, 1983; Heston *et al.*, 1986). Within the US, rates among boys ages 0–4 in Connecticut were lower in the past and higher during recent years than those in the larger population in the five geographic areas (Heston *et al.*, 1986; Devesa *et al.*, 1987). Acute leukaemia incidence among children ages 0–9 increased in Denmark until about 1970, with declines thereafter (Hansen *et al.*, 1983). Leukaemia rose among boys ages 0–4 in Great Britain over the period 1968–76 (Stiller & Draper, 1982), but rates were still lower than those observed in the five US geographic areas. The dramatic decline in US childhood leukaemia mortality since 1950 among the youngest children and since the mid-to-late 1960s among older children (see also Steinhorn & Ries, 1988) is primarily due to treatment advances for acute lymphocytic leukaemia (Pinkel, 1987).

Limitations of the data include differences in methods for case ascertainment and validation (particularly in cell-type specification) among registries (Alexander *et al.*, 1989), variation in the proportion of other and NOS, and the relative frequencies of ALL and ANLL within the other and NOS category. Errors may occur in the intercensal estimates due to high mobility and/or substantial illegal immigration. Furthermore, findings based on small numbers should be considered as tentative until additional cases substantiate a clear pattern.

Ideally, state-of-the-art characterisation of childhood leukaemia cases should be used (Greaves *et al.*, 1985) to clarify and compare patterns of ALL and ANLL subtype occurrence among populations. The discordance in sex ratios, age-specific differences, and the low rates of ALL among US blacks compared to whites warrant more detailed evaluation. The consistent finding of a peak incidence at ages 1–4 for ALL suggests that prenatal in conjunction with early infancy-related exposures ought to continue to be an important focus in analytic studies. Special opportunities are provided by the differing patterns of childhood leukaemia among racial/ethnic groups in Kuwait and Israel, and the high rates in Costa Rica and some groups in Hawaii, although small numbers may be a limiting factor. Finally, similar methodologies should be used across studies in assessing specific risk factors to facilitate comparisons.

We thank Mr Vladimir Dragunsky and Mr Jim Abbott of Information Management Services, Inc. for computer programming and graphics support.

### References

ALEXANDER, F., RICKETTS, T.J., McKINNEY, P.A. & CARTWRIGHT, R.A. (1989). Cancer registration of leukaemias and lymphomas: results of a comparison with a specialist registry. *Comm. Med.*, **11**, 89.

BERMUDEZ, D.G. (1985). The National Tumor Registry in Costa Rica. *Epidemiol. Bull. Pan Am. Health Org.*, **6**, 10.

BESSHO, F. (1989). Acute non-lymphocytic leukemia is not a major type of childhood leukemia in Japan. *Eur. J. Cancer Clin. Oncol.*, **25**, 729.

BOWIE, C. (1987). The validity of a cancer register in leukemia epidemiology. *Comm. Med.*, **9**, 152.

BOWMAN, W.P., PRESBURY, G., MELVIN, S.L., GEORGE, S.L. & SIMONE, J.W. (1984). A comparative analysis of acute lymphocytic leukemia in white and black children: presenting clinical features and immunological markers. In: *Pathogenesis of Leukemias and Lymphomas: Environmental Influences*, Magrath, I.T., O'Conor, G.T., Ramot, B. (eds), p. 169. Raven Press: New York.

BUCKLEY, J.D., ROBISON, L.L., SWOTINSKY, R. & 8 others (1980). Occupational exposures of parents of children with acute non-lymphocytic leukemia: a report from the Children's Cancer Study Group. *Cancer Res.*, **49**, 4030.

BUTTARINI, A. & GALE, R.P. (1989). Age of onset and type of leukaemia. *Lancet*, **ii**, 789.

CELADE (Centro Latinoamericano de Demografia) (1983). Proyeccion de poblacion. Revisado en 1982. Interpretation por anos, calendario, San Jose, Costa Rica.

COURT BROWN, W.M. & DOLL, R. (1961). Leukaemia in childhood and young adult life. Trends in mortality in relation to aetiology. *Br. Med. J.*, **26**, 981.

DEVESA, S.S., SILVERMAN, D.T., YOUNG, J.L. Jr & 7 others (1987). Cancer incidence and mortality trends among whites in The United States, 1947-84. *J. Natl Cancer Inst.*, **79**, 701.

DOLL, R. & SMITH, P.G. (1982). Comparison between registries: age-standardized rates. In: *Cancer Incidence in Five Continents Vol IV*, Waterhouse, J., Muir, C.S., Shanmugaratnam, K., Powell, J. (eds), p. 671. IARC Scientific Publication 42: Lyon.

DUNCAN, M.H., WIGGINS, C.L., SAMET, J.M. & KEY, C.R. (1986). Childhood cancer epidemiology in New Mexico's American Indians, Hispanic whites, and non-Hispanic whites, 1970-82. *J. Natl Cancer. Inst.*, **76**, 1013.

FLEMING, A.S. (1985). The epidemiology of lymphomas and leukemias in Africa-an overview. *Leuk. Res.*, **9**, 735.

FRAUMENI, J.F. Jr & MILLER, R.W. (1967). Epidemiology of human leukemia: recent observations. *J. Natl Cancer Inst.*, **38**, 593.

GARDNER, M.J., SNEE, M.P., HALL, A.J., POWELL, C.A., DOWNES, S. & TERRELL, J.D. (1990). Results of a case-control study of leukaemia and lymphoma among young people near Sellafield nuclear plant in West Cumbria. *Br. Med. J.*, **300**, 423.

GOODMAN, M.T., YOSHIZAWA, C.N. & KOLONEL, L.N. (1989). Incidence trends and ethnic patterns for childhood leukaemia in Hawaii: 1960-1984. *Br. J. Cancer*, **60**, 93.

GREAVES, M.F., PEGRAM, S.M. & CHAN, L.C. (1985). Collaborative group study of the epidemiology of acute lymphocytic leukemia subtypes: background and first report. *Leuk. Res.*, **6**, 715.

GREAVES, M.F. (1989). Etiology of childhood acute lymphoblastic leukemia: a soluble problem? In: *Acute Lymphoblastic Leukemia.* UCLA Symposium on Molecular and Cellular Biology, New Series Vol 108. Gale, R.P., Hoelzer, D. (eds). Alan Liss Inc.: New York.

GREENBERG, R.S. & SCHUSTER, J.L. Jr (1985). Epidemiology of cancer in children. *Epidemiol. Rev.*, **7**, 22.

HANSEN, N.E., KARLE, H. & JENSEN, O.M. (1983). Trends in the incidence of leukemia in Denmark, 1943-1977: an epidemiologic study of 14,000 patients. *J. Natl Cancer Inst.*, **71**, 697.

HESTON, J.F., KELLY, J.B., MEIGS, J.W. & FLANNERY, J.T. (1986). Forty-five years of cancer incidence in Connecticut: 1935-79. *Natl Cancer Inst Monogr.*, **70**, 506.

KINLEN, L. (1988). Evidence for an infective cause of childhood leukaemia: comparison of a Scottish New Town with nuclear reprocessing sites in Britain. *Lancet*, **ii**, 1323.

LOWENGART, R.A., PETERS, J.,M., CICIONI, C. & 4 others (1987). Childhood leukemia and parents occupational and home exposures. *J. Natl Cancer Inst.*, **79**, 781.

MACMAHON, B. (1962). Prenatal X-ray exposure and childhood cancer. *J. Natl Cancer Inst.*, **28**, 1173.

MATA, L. & ROSERO, L. (1988). National Health and Social Development in Costa Rica: a case study of intersectoral action. Technical Paper No. 13. Pan American Sanitary Bureau, Pan American Health Organization: Washington, D.C.

MENCK, H.R. & ROSS, R.K. (1988). A county-wide (seven ethnicity) population model for Los Angeles County, 1970-1985, CSP Technical Report No. 1. Cancer Surveillance Program of Los Angeles County, Los Angeles.

MILLER, R.W. & DALAGER, N.A. (1974). US childhood cancer deaths by cell type, 1960-68. *Pediat.*, **85**, 664.

NATIONAL CANCER INSTITUTE (1989). Cancer Statistics Review 1973-1986, including a Report on The Status of Cancer Control. Bethesda, MD: NCI, NIH Publication No. 89-2789.

NEGLIA, J.P. & ROBISON, L.L. (1988). Epidemiology of childhood acute leukemias. *Ped. Clin. N. Am.*, **35**, 675.

PARKIN, D.M., STILLER, C.A., DRAPER, G.J., BIEBER, C.A., TERRACINI, B. & YOUNG, J.L. (1988). International Incidence of Childhood Cancer. IARC Scientific Publication 87: Lyon.

PINKEL, D. (1987). Curing children of leukemia. *Cancer*, **59**, 1683.

RAMOT, B. & MAGRATH, I. (1982). Hypothesis: the environment is a major determinant of the immunologic subtype of lymphoma and acute lymphoblastic leukaemia. *Br. J. Haematol.*, **52**, 183.

SAVITZ, D.A., WACHTEL, H., BARNES, F.A., JOHN, E.M. & TURDIK, J.G. (1988). Case-control study of childhood cancer and exposure to 60 Hz magnetic fields. *Am. J. Epidemiol.*, **45**, 543.

SHU, X.-O., GAO, Y.-T., LINET, M.S. & 4 others (1987). Chloramphenicol use and childhood leukemia in Shanghai. *Lancet*, **ii**, 934.

SIERRA, R., PARKIN, D.M., BARRANTES, R., BIEBER, C.A., MUNOZ, G. & MUNOZ, N. (1988). Cancer in Costa Rica. Technical Report. 1 IARC: Lyon.

SIERRA, R., PARKIN, D.M. & LEIVA, G.M. (1989). Cancer in Costa Rica. *Cancer Res.*, **49**, 717.

STEINHORN, S.C. & RIES, L.G. (1988). Improved survival among children with acute leukemia in the United States. *Biomed. & Pharmacother.*, **42**, 675.

STEWART, A., WEBB, J. & MERITT, D. (1958). A survey of childhood malignancies. *Br. Med. J.*, **2**, 1495.

STILLER, C.A. & DRAPER, G.J. (1982). Trends in childhood leukemia in Britain, 1968–1978. *Br. J. Cancer*, **45**, 543.

TOLLERUD, D.J., BROWN, L.M., BLATTNER, W.A. & HOOVER, R. (1990, in press). The influence of race on T-cell subsets. In: *Epidemiology and Biology of Multiple Myeloma*. Potter, M., Obrams, G.I. (eds). Springer-Verlag: Berlin.

WILLIAMS, C.K.O., FOLAMI, A.O., LADITAN, A.A.O. & UKAEJIOFO, E.O. (1982). Childhood acute leukemia in a tropical population. *Br. J. Cancer*, **46**, 89.

YOUNG, J.L. Jr, PERCY, C.L., ASIRE, A.J. (eds) (1981). Surveillance, Epidemiology, and End Results: Incidence and Mortality Data, 1973–77. *Natl Cancer Inst. Monogr.*, **57**, 1.